# Instantaneous Ambiguity Reinitialization and Fast Ambiguity Initialization for L1-L2 GPS Measurements

**DOI:** 10.3390/s20205730

**Published:** 2020-10-09

**Authors:** Mieczysław Bakuła

**Affiliations:** Institute of Navigation, Military University of Aviation, 08-521 Dęblin, Poland; mieczyslaw.bakula@gmail.com

**Keywords:** ambiguity resolution, L1-L2, GPS, GNSS, PREFMAR

## Abstract

This paper presents a **PRE**cise and **F**ast **M**ethod of **A**mbiguity **R**einitialization/**R**esolution (PREFMAR) for L1 and L2 in GPS measurements. The method determines $N_{L1}$ and $N_{L2}$ ambiguities based on the ambiguity functions: $\Psi {\left(N_{L1}\right)}_{N_{L1}N_{L2}}$ and $\Psi {\left(N_{L2}\right)}_{N_{L2}N_{L1}}$. These ambiguity functions have been described in detail in this work. The developed method of ambiguity initialization and reinitialization in relative positioning can use Global Positioning System (GPS) measurements from only two satellites and one measurement epoch. To resolve $N_{L1}$ and $N_{L2}$ ambiguities, a variance-covariance (VC) matrix of the float solution is not needed. The size of the search area in the PREFMAR method depends on code and phase accuracy as well as on the GNSS signal frequencies. Therefore, the search area is specific for every double or triple Global Navigation Satellite Systems (GNSS) data frequency. However, this part of the research only presents the ambiguity search area for L1 and L2 of GPS measurements. Additionally, a numerical example has been analyzed in detail with the use of the PREFMAR method and a float solution (${\tilde{N}}_{L1}$, ${\tilde{N}}_{L2}$). Finally, the elaborated algorithms were successfully tested on real L1 and L2 GPS measurements for instantaneous ambiguity reinitialization. The PREFMAR method allows instantaneous ambiguity reinitialization if all satellites lose contact with a GNSS antenna, for short and long baselines. Therefore, the PREFMAR has a great potential for precise real-time kinematic GNSS navigation.

## 1. Introduction

Attempts to determine the total ambiguities in phase measurements in GPS observations date back to the late 1970s—i.e., GPS satellites were first placed in Earth orbits in 1978 [1,2]. The first research paper to appear on GPS measurement and determination of ambiguities in phase measurements for GPS observations was published by Counselman and Gourevitch [3]. With the initially quite weak constellation of GPS satellites, it was already proved to achieve precision levels down to centimeters for baselines ca. 10 km long. This was performed during static sessions of 2-3 h [4]. Even though three decades have elapsed since then, the issue of ambiguity determination is still subject to intense research and development work in numerous research centers all over the world. This has resulted in various methods applicable not only to statistical measurements but also to kinematic measurements, including those performed in real-time. Kim and Langley [5] provide a general outline on methods of ambiguity resolution developed in the period between 1981 and 1999 where ambiguity resolution methods were divided into three main groups: ambiguity search in the measurement domain, search in the coordinate domain and search in the ambiguity domain. Such divisions still remain valid and correspond well to the current methods. The first group is based on linear combinations of GNSS observations. The linear combinations presented by Melbourne [6] or Wubbena [7] were originally invented by Hatch [8]. This approach uses dual frequency of code and carrier phase measurements to fixed ambiguities for different integer lineal combinations. Many ambiguity resolution strategies resolve firstly the fundamental Melbourne-Wubbena-Hatch combination-the so-called wide-line linear combination. Once the wide-lane ambiguity is resolved, then the L1 and L2 integer ambiguity solutions are easier performed [9]. The second method is based on the mathematical ambiguity function proposed by Counselman and Gourevitch (1981) and further investigated by Remondi [10]. Han and Rizos [11] improved computational efficiency of the ambiguity function algorithms for practical applications. Cellmer et al. [12] proposed further improvement of the ambiguity function approach. They have developed a new method named the Modified Ambiguity Function Approach (MAFA), in which the dramatically reduced computational load characterizes the computational process. The third group is currently the most popular and is based on estimation with the integer least-squares principle. The least-squares ambiguity search solution is an approximate solution with a description of the volume (e.g., ellipsoid) over which the search is to be conducted [13]. The start-up procedure was based on the differential code solution for the initial position, and the search was performed over the associated three-sigma region surrounding that position. Based on numerical tests performed by Hatch, a single-epoch solution was possible under the following conditions: (1) dual frequency data are available, (2) distances are limited to a few tens and the ionospheric refraction effect is not too severe, (3) seven or more total satellites are available for processing with good geometry. This was revolutionary research that shows that a precise kinematic surveying or precise navigation is possible when L1 and L2 GPS measurements are available. Frei and Beutler [14] introduced an efficient algorithm of Fisher’s probability density function to form confidence regions for individual ambiguities between float (real numbers) and fixed (integer) solutions for validation of the final fixed ambiguities. Teunissen improved the idea presented by Hatch by the use of variance covariance matrix of the float solution to define the search area [15]. The search area is represented by an ellipsoid, with orientation dependent on the variance covariance matrix. The LAMBDA method is the most popular and famous method in GNSS world. The results of the PREFMAR approach can be compared with the LAMBDA solution, because a single epoch solution was presented in detail based on the float solution. All of these groups can be combined and modified to improve efficiency and reliability, especially for the integration of more than one GNSS system. The current research of ambiguity resolution is rather focused on triple-frequencies. The review of triple-frequency ambiguity resolutions was presented in [16,17]. Numerical comparisons of multicarrier ambiguity resolution for relative GNSS positioning was investigated by O’Kneefe et al. [18]. 

The combination of L1-L2 frequencies of a GPS system is a special one, available in every multi-frequency GNSS receiver and probably the most effective double-frequency combination of GNSS data. A detailed description of mathematical functions used in the PREFMAR approach was investigated earlier for the use of L1-L5 or E1-E5a GPS/GALILEO data [19], but each double combination of GNSS frequencies must be carefully analyzed to find specific properties for fast and precise ambiguity initialization and reinitialization. Therefore, this paper is devoted to the most important combination in GNSS frequencies—i.e., L1-L2 GPS measurements. This research is focused not only on the initialization of ambiguities but also presents innovative method for instantaneous reinitialization of ambiguities for L1-L2 measurements. Both initialization and reinitialization of ambiguities are realized based on the new mathematical functions: $\Psi {\left(N_{L1}\right)}_{N_{L1}N_{L2}}$ and $\Psi {\left(N_{L2}\right)}_{N_{L2}N_{L1}}$. A mathematical background of the function is based on a strong correlation between float solutions (see Section 2, Section 3 and Section 4). In Section 5, properties of these functions are discussed in terms of real relative errors of the double difference carrier phase and pseudorange of L1 and L2 GPS measurements. These properties were discovered by the author during analysis of real GPS data. Therefore, Section 5 presents different templates for ambiguity initialization in terms of magnitude of relative carrier phase and code errors. Section 6 presents a detailed numerical example for a single epoch were a float GPS L1-L2 solution is available. Section 7 analyses the efficiency of ambiguity resolution for the 3.6 km GPS baseline. Summary and conclusions of the research are given in Section 8.

## 2. Double-Differenced GPS Data Equations and the Correlation of Geometry-Free Ambiguities

Let us make double-difference equations for two satellites, two measurement points and two frequencies: L1 and L2. For a very short baseline, the ionospheric and tropospheric delays have been ignored. Then, for the two frequencies, double-difference (DD) equations for carriers (ϕ) and pseudo-ranges (P) can be written as follows:

(1) $$\lambda_{L1}\phi_{L1}\left(t\right)=\varrho \left(t\right)+\lambda_{L1}N_{L1}+\lambda_{L1}\varepsilon_{\phi_{L1}}\left(t\right)$$

(2) $$P_{L1}\left(t\right)=\varrho \left(t\right)+\varepsilon_{P_{L1}}\left(t\right)$$

(3) $$\lambda_{L2}\phi_{L2}\left(t\right)=\varrho \left(t\right)+\lambda_{L2}N_{L2}+\lambda_{L2}\varepsilon_{\phi_{L2}}\left(t\right)$$

(4) $$P_{L2}\left(t\right)=\varrho \left(t\right)+\varepsilon_{P_{L2}}\left(t\right)$$

where:

$\phi_{L1},\phi_{L2}$—observations of double-difference phase measurements for L1 and L2 frequencies (in cycles); ϱ(t)—double-difference geometric range [m]; $\lambda_{L1}=$0.190293672798365 [m]; $\lambda_{L2}=$ 0.244210213424568 [m].

The above equations can be also presented in a matrix notation AX=L, as [20]:

(5) $$\left[\begin{array}{cc} 1 & \;\;\begin{array}{cc} \lambda_{L1} & 0 \end{array}\\ \begin{array}{c} 1\\ \begin{array}{c} 1\\ 1 \end{array} \end{array} & \;\;\;\begin{array}{cc} \begin{array}{c} 0\\ \begin{array}{c} 0\\ 0 \end{array} \end{array} & \begin{array}{c} \;\;\;0\\ \begin{array}{c} \lambda_{L2}\\ \;\;\;0 \end{array} \end{array} \end{array} \end{array}\right]\left[\begin{array}{c} \varrho \left(t\right)\\ \begin{array}{c} N_{L1}\\ N_{L2} \end{array} \end{array}\right]=\left[\begin{array}{c} \lambda_{L1}\phi_{L1}\left(t\right)\\ \begin{array}{c} P_{L1}\left(t\right)\\ \begin{array}{c} \lambda_{L2}\phi_{L2}\left(t\right)\\ P_{L2}\left(t\right) \end{array} \end{array} \end{array}\right]$$

where the *X* vector of unknowns represents the $N_{L1}$ and $N_{L2}$ ambiguities, and the ϱ(t) value. The $N_{L1}$ and $N_{L2}$ integer values remain invariable in time if a GPS antenna has no interruption in the reception of phase signals. 

By examining the L1 and L2 frequencies (Equations (1)–(4) separately, we can calculate $N_{L1}$ and $N_{L2}$ ambiguities for a single measurement epoch:

(6) $$N_{L1}={\left[\phi_{L1}\left(t\right)-\frac{P_{L1}\left(t\right)}{\lambda_{L1}}\right]}_{\mathrm{roundoff}}$$

(7) $$N_{L2}={\left[\phi_{L2}\left(t\right)-\frac{P_{L2}\left(t\right)}{\lambda_{L2}}\right]}_{\mathrm{roundoff}}$$

If thus, the accuracy of $P_{L1}\left(t\right)$ and $P_{L2}\left(t\right)$ code measurements would be better than 0.5 of the length of respective waves, then using formulas 6 and 7, we could precisely calculate the $N_{L1}$ and $N_{L2}$ ambiguities by rounding to the nearest integer. Unfortunately, the accuracy of code measurements is far worse, which makes it impossible to calculate $N_{L1}$ and $N_{L2}$ ambiguities in a reliable manner.

To achieve a strong correlation between the $N_{L1}$ and $N_{L2}$ values, a common (ω) quantity, which would pertain to code measurements in specific measurement epochs (t), needs to be introduced to the equations above:

(8) $$N_{L1}=\phi_{L1}\left(t\right)-\frac{\omega \left(t\right)}{\lambda_{L1}}$$

(9) $$N_{L2}=\phi_{L2}\left(t\right)-\frac{\omega \left(t\right)}{\lambda_{L2}}$$

where:

(10) $$\omega \left(t\right)=\varrho_{o}\left(t\right)$$

or [21]

(11) $$\omega \left(t\right)=0.5*\left[P_{L1}\left(t\right)+P_{L2}\left(t\right)\right]$$

Although the value ω(t) can be calculated using only a double-difference (DD) observation for a pair of satellites (Equation (11)), the most accurate ω(t) is obtained using double differenced geometric distance (Equation (10)) based on code relative or differential GNSS positioning based on Kalman filter and network code DGNSS solutions [22,23]. 

**Figure 1:**
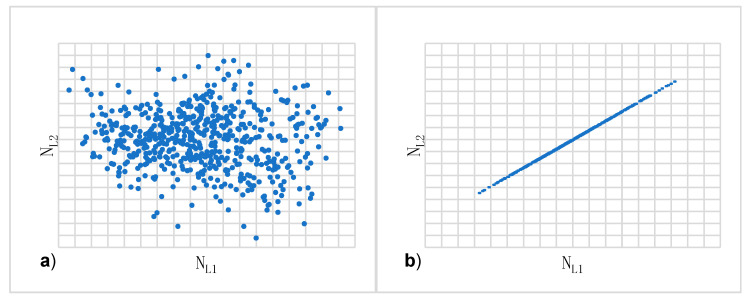
Scatter plot of uncorrelated (**a**) and correlated (**b**) geometry-free $N_{L1}$ and $N_{L2}\;$ ambiguities.

In Figure 1 that the $N_{L1}$ and $N_{L2}$ observations are strongly correlated, as the $N_{L1}$ and $N_{L2}$ values are located precisely along a straight line (Figure 1b). The value of the correlation coefficient between $\;N_{L1}$ and $N_{L2}$ ambiguities for data included in Figure 1b is equal to 0.999946. The data presented pertain to a vector with a length of 3.6 km and the use of two GPS receivers - Ashtech Z-XII and Topcon Hiper Pro - and based only on two satellites.

## 3. Ambiguity Regression Line Equations for L1-L2 Measurements

Regression line equation for correlated $N_{L2}$ ambiguities with reference to $N_{L1}$ ambiguity in the $N_{L1}N_{L2}$ system of ambiguities can be written with the following equation:

(12) $$N_{L2}=a_{L1,L2}N_{L1}+b_{L1,L2}$$

or

(13) $${\tilde{N}}_{L2}=a_{L1,L2}{\tilde{N}}_{L1}+b_{L1,L2}$$

where:

(14) $$a_{L1,L2}=\frac{f_{L2}}{f_{L1}}=\frac{\lambda_{L1}}{\lambda_{L2}}=\frac{60}{77}=0.779220779220779,$$

$f_{L1}=1575.42\;\left[\mathrm{MHz}\right]$; $f_{L2}=1227.60\;\left[\mathrm{MHz}\right]$.

As ambiguities in the form of real numbers (${\tilde{N}}_{L1},\;{\tilde{N}}_{L2}$) lie along a straight line expressed with the Equation (13), the $b_{L1,L2}$ value can be calculated with the following formula

(15) $$b_{L1,L2}={\tilde{N}}_{L2}-\frac{60}{77}{\tilde{N}}_{L1}$$

thus

(16) $$b_{L1,L2}=\left(\phi_{L2}-\frac{\omega }{\lambda_{L2}}\right)-\frac{60}{77}\left(\phi_{L1}-\frac{\omega }{\lambda_{L1}}\right).$$

Based on the Equation (16), it can be seen that the $b_{L1,L2}$ value can be calculated for a single measurement epoch, without knowing integer $N_{L1}$ and $N_{L2}$ values. While the Equation (12) allows the determination of the integer $N_{L2}$ value if the integer $N_{L1}$ value is known. If $N_{L2}$ is known, after transforming the Equation (12), we may calculate the $N_{L1}$ value:

(17) $$N_{L1}=\frac{77}{60}\left(N_{L2}-b_{L1,L2}\right)$$

Finally, for DD observations, the regression line equation in the $N_{L1}N_{L2}$, system for a single measurement epoch can be written as:

(18) $${\tilde{N}}_{L2}=\frac{60}{77}N_{L1}+b_{L1,L2}=\;\frac{60}{77}N_{L1}+\left(\phi_{L2}-\frac{\omega }{\lambda_{L2}}\right)-\frac{60}{77}\left(\phi_{L1}-\frac{\omega }{\lambda_{L1}}\right).$$

whereas for n measurement epochs, we have a detailed formula determining the relation between integer $N_{L1}$ values and calculated (real) ${\tilde{N}}_{L2}$ values. Based on this we receive:

(19) $${\tilde{N}}_{L2}=\frac{60}{77}N_{L1}+n^{-1}\overset{n}{\underset{i=1}{\sum }}\left(\phi_{L2,i}-\frac{\omega_{i}}{\lambda_{L2}}\right)-n^{-1}\frac{60}{77}\overset{n}{\underset{i=1}{\sum }}\left(\phi_{L1,i}-\frac{\omega_{i}}{\lambda_{L1}}\right)$$

or in simplified form

(20) $${\tilde{N}}_{L2}=a_{L1,L2}N_{L1}+n^{-1}\sum_{i=1}^{n}{\tilde{N}}_{L2,i}-n^{-1}a_{L1,L2}\sum_{i=1}^{n}{\tilde{N}}_{L1,i}.$$

## 4. Ambiguity Functions for L1-L2 GPS Measurements 

Assuming that the $b_{L1,L2}$ values are equal to zero, for distribution of the i  set of ambiguities being integer numbers in the $N_{L1}N_{L2}$ system, we can write

(21) $$N_{L2,i}=a_{L1,L2}N_{L1,i}+b_{L1,L2}=a_{L1,L2}N_{L1,i}=\frac{60}{77}N_{L1,i}$$

However, in the majority of cycles, the calculated $N_{L2,i}$ value with reference to the integer $N_{L1,i}\;$ value is a real number, thus

(22) $${\tilde{N}}_{L2,i}=a_{L1,L2}N_{L1,i}=\frac{60}{77}N_{L1,i}$$

Analogically, for the $N_{L2}N_{L1}$ system, we have:

(23) $$N_{L1,i}=a_{L1,L2}{}^{-1}N_{L2,i}=\frac{77}{60}\;N_{L2,i}$$

and

(24) $${\tilde{N}}_{L1,i}=a_{L1,L2}{}^{-1}N_{L2}=\frac{77}{60}\;N_{L2,i}$$

For Equations (22) and (24), we can determine the value of certain ε error in the following manner:

(25) $$\varepsilon_{L1,L2}={\tilde{N}}_{L2}-{\left[{\tilde{N}}_{L2}\right]}_{\mathrm{roundoff}}$$

or

(26) $$\varepsilon_{L2,L1}={\tilde{N}}_{L1}-{\left[{\tilde{N}}_{L1}\right]}_{\mathrm{roundoff}}$$

meeting the relation $\varepsilon_{L1,L2}=-\varepsilon_{L2,L1}$, as these are relative errors in phase observations (DD).

Then, in the Formula (25), we substitute for ${\tilde{N}}_{L2}$ the expression from the Formula (18), thus receiving the following function $\Psi {\left(N_{L1}\right)}_{N_{L1}N_{L2}}$ = $\varepsilon_{L1,L2}$ (in cycles) for the $N_{L1}N_{L2}$ system.

(27) $$\begin{array}{cc} \Psi {\left(N_{L1}\right)}_{N_{L1}N_{L2}}= & \lambda_{L2}\left({\tilde{N}}_{L2}-{\left[{\tilde{N}}_{L2}\right]}_{\mathrm{roundoff}}\right)\\ & =\lambda_{L2}(\frac{60}{77}N_{L1}+\left(\phi_{L2}-\frac{\omega }{\lambda_{L2}}\right)-\frac{60}{77}\left(\phi_{L1}-\frac{\omega }{\lambda_{L1}}\right)\\ & -{\left[\frac{60}{77}N_{L1}+\left(\phi_{L2}-\frac{\omega }{\lambda_{L2}}\right)-\frac{60}{77}\left(\phi_{L1}-\frac{\omega }{\lambda_{L1}}\right)\right]}_{\mathrm{roundoff}}) \end{array}$$

Analogically, for the $N_{L2}N_{L1}$ system and the function $\Psi {\left(N_{L2}\right)}_{N_{L2}N_{L1}}$ = $\varepsilon_{L2,L1}$, where $\varepsilon_{L2,L1}=-\varepsilon_{L1,L2}$

(28) $$\begin{array}{cc} \Psi {\left(N_{L2}\right)}_{N_{L2}N_{L1}}= & \lambda_{L1}\left({\tilde{N}}_{L1}-{\left[{\tilde{N}}_{L1}\right]}_{\mathrm{roundoff}}\right)\\ & =\lambda_{L1}(\frac{77}{60}N_{L2}+\left(\phi_{L1}-\frac{\omega }{\lambda_{L1}}\right)-\frac{77}{60}\left(\phi_{L2}-\frac{\omega }{\lambda_{L2}}\right)\\ & -{\left[\frac{77}{60}N_{L2}+\left(\phi_{L1}-\frac{\omega }{\lambda_{L1}}\right)-\frac{77}{60}\left(\phi_{L2}-\frac{\omega }{\lambda_{L2}}\right)\right]}_{\mathrm{roundoff}}) \end{array}$$

**Figure 2:**
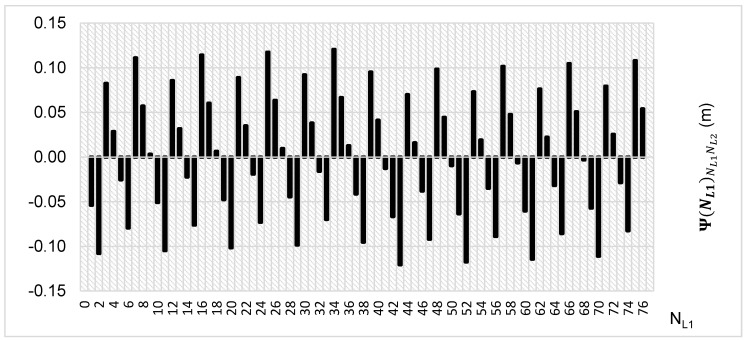
Behavior of a full period of the $\Psi {\left(N_{L1}\right)}_{N_{L1}N_{L2}}$ function in the $N_{L1}N_{L2}$ system, in the $N_{L1}\subset \langle 0;77\rangle$ interval, for GPS observations of L1 and L2 frequencies.

**Figure 3:**
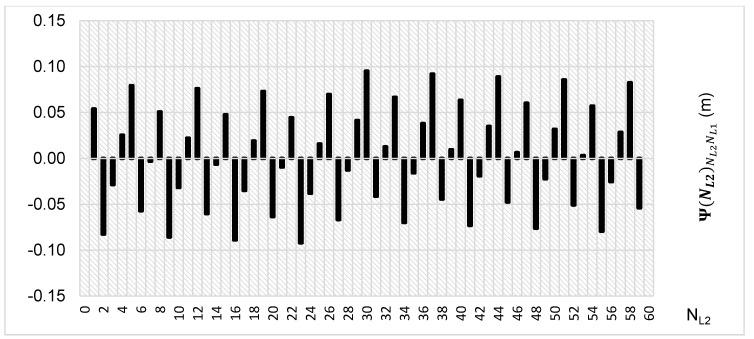
Behavior of a full period of the $\Psi {\left(N_{L2}\right)}_{N_{L2}N_{L1}}$ function in the $N_{L2}N_{L1}$ system, in the $N_{L2}\subset \langle 0;60\rangle$ interval, for GPS observations L1 and L2 frequencies.

**Figure 4:**
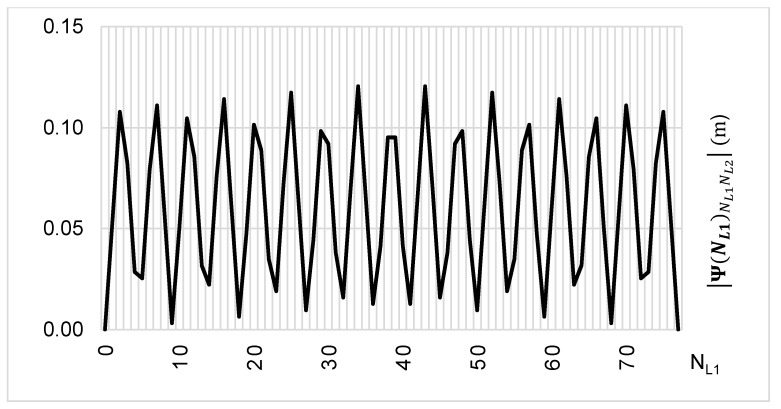
Behavior of a full period of the $\left|\Psi {\left(N_{L1}\right)}_{N_{L1}N_{L2}}\right|$ function in the $N_{L1}N_{L2}$ system, in the $N_{L1}\subset \langle 0;77\rangle$ interval, for GPS observations of L1 and L2 frequencies.

**Figure 5:**
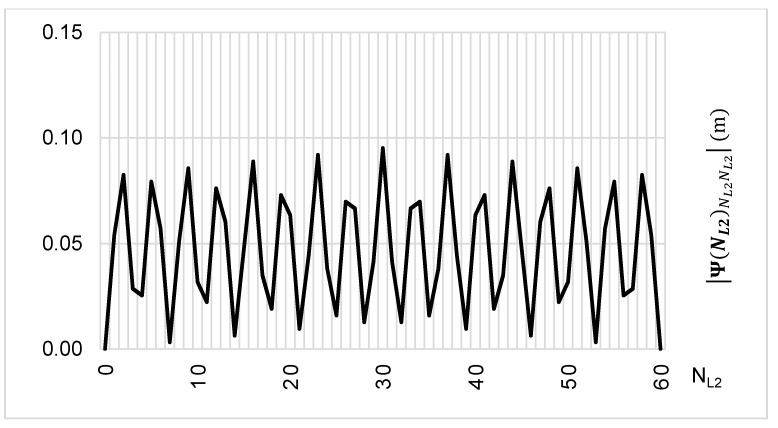
Behavior of a full period of the $\left|\Psi {\left(N_{L2}\right)}_{N_{L2}N_{L1}}\right|$ function in the $N_{L2}N_{L1}$ system, in the $N_{L2}\subset \langle 0;60\rangle$ interval, for GPS observations of L1 and L2 frequencies.

The above-presented functions are used in the PREFMAR method. Behaviors of the $\Psi {\left(N_{L1}\right)}_{N_{L1}N_{L2}}$ and $\Psi {\left(N_{L2}\right)}_{N_{L2}N_{L1}}$ functions for a $b_{L1,L2}$ value equal to zero have been presented in Figure 2 and Figure 3. Additionally, behaviours of the $\left|\Psi {\left(N_{L1}\right)}_{N_{L1}N_{L2}}\right|$ and $\left|\Psi {\left(N_{L2}\right)}_{N_{L2}N_{L1}}\right|$ functions have been presented in Figure 4 and Figure 5, where that their minima and thus their periodic character (repeatability) can clearly be seen. Based on an analysis of the values of the $\Psi {\left(N_{L1}\right)}_{N_{L1}N_{L2}}$ functions lying in the (−0.5$\lambda_{L2}$; 0.5 $\lambda_{L2}$) interval, it can be observed that the value of the $\Psi {\left(N_{L1}\right)}_{N_{L1}N_{L2}}$ function for $f_{L1}$ and $f_{L2}$ frequencies of GPS observations repeat precisely every 77 $N_{L1}$ cycles. Additionally, the $\left|\Psi {\left(N_{L1}\right)}_{N_{L1}N_{L2}}\right|$ function (Figure 4) has its minima also every 77 cycles—i.e.,

(29) $$\left|\Psi {\left(N_{L1}=0\pm 77\right)}_{N_{L1}N_{L2}}\right|=min=0$$

To the contrary, based on an analysis of the values of $\Psi {\left(N_{L2}\right)}_{N_{L2}N_{L1}}$, that analogically as for the $\Psi {\left(N_{L1}\right)}_{N_{L1}N_{L2}}$ function are comprised in the (-0.5$\lambda_{L1}$; 0.5 $\lambda_{L1}$) interval, the values of the $\Psi {\left(N_{L2}\right)}_{N_{L2}N_{L1}}$ function for $f_{L1}$ and $f_{L2}$ frequencies of GPS observations repeat precisely every 60 $N_{L2}$ cycles. Additionally, the $\left|\Psi {\left(N_{L2}\right)}_{N_{L2}N_{L1}}\right|$ function (Figure 5) has its minima also every 60 cycles—i.e.,

(30) $$\left|\Psi {\left(N_{L2}=0\pm 60\right)}_{N_{L2}N_{L1}}\right|=min=0$$

when analyzing the behavior of the $\Psi {\left(N_{L1}\right)}_{N_{L1}N_{L2}}$ and $\Psi {\left(N_{L2}\right)}_{N_{L2}N_{L1}}$ functions, it can be seen that both functions have the same wavelength and frequency:

(31) $$\lambda_{\Psi {\left(N_{L1}\right)}_{N_{L1}N_{L2}}}\;=\;\lambda_{\Psi {\left(N_{L2}\right)}_{N_{L2}N_{L1}}}\;=\;14.6526128054741\;m$$

(32) $$f_{\Psi {\left(N_{L1}\right)}_{N_{L1}N_{L2}}}\;=\;f_{\Psi {\left(N_{L2}\right)}_{N_{L2}N_{L1}}}\;=\;20.46\;\mathrm{MHz}$$

because

(33) $$77\times \lambda_{L1}=60\times \lambda_{L2}$$

and they are equivalent in the process of determining $N_{L1}$ and $N_{L2}$ ambiguities for the value of

(34) $$\left|\varepsilon_{L1,L2}\right|\leq 0,5\lambda_{L1}\leq 0.0951468363991824\;m$$

## 5. Ambiguity Search Space in the PREFMAR Method 

When analyzing the ambiguity functions of the PREFMAR method, formulated based on correlated $N_{L1}$ and $N_{L2}$ observations, certain $\varepsilon_{L1,L2}$ (Equations (25) and (26)) values need to be considered as relative errors in L1 and L2 phase observations that will help us to understand interpretations of the $\Psi {\left(N_{L1}\right)}_{N_{L1}N_{L2}}$ and $\Psi {\left(N_{L2}\right)}_{N_{L2}N_{L1}}$ functions. Thus, let us write

(35) $$\lambda_{L2}\varepsilon_{\phi \left(L2\right)}-\lambda_{L1}\varepsilon_{\phi \left(L1\right)}=\varepsilon_{L1,L2}$$

(36) $$\lambda_{L1}\varepsilon_{\phi \left(L1\right)}-\lambda_{L2}\varepsilon_{\phi \left(L2\right)}=\varepsilon_{L2,L1}$$

If errors in DD phase observations for $f_{L1}$ and $f_{L2}$ frequencies of GPS observations are equal and have the same value, then:

(37) $$\varepsilon_{\phi \left(L2\right)}-\varepsilon_{\phi \left(L1\right)}=0$$

Thus, if $\varepsilon_{L1,L2}=-\varepsilon_{L2,L1}=0,$ then the minima of the $\left|\Psi {\left(N_{L1}\right)}_{N_{L1}N_{L2}}\right|$ and $\left|\Psi {\left(N_{L2}\right)}_{N_{L2}N_{L1}}\right|$ functions indicate the searched values of $N_{L1}$ and $N_{L2}$ ambiguities. The question remains, however, how large the errors in DD phase observations can be for the ambiguities to be determined by the ambiguity function minima, assuming the Equation (37). The answer to this question depends on the smallest value of the functions expressed in the Equations (27) and (28) and, in the case of L1–L2 phase measurements of GPS observations, these are the $\Delta_{\Psi {\left(N_{L1}\right)}_{N_{L1}N_{L2}}}$ and $\Delta_{\Psi {\left(N_{L2}\right)}_{N_{L2}N_{L1}}}$ values equal to

(38) $$\Delta_{\Psi {\left(N_{L1}\right)}_{N_{L1}N_{L2}}}=\Delta_{\Psi {\left(N_{L2}\right)}_{N_{L2}N_{L1}}}=\frac{1}{77}\lambda_{L2}=\frac{1}{60}\lambda_{L1}=0.00317156121330608\;m$$

The 38 formula thus determines the smallest unit of the $\Psi {\left(N_{L1}\right)}_{N_{L1}N_{L2}}$ function in the $N_{L2}N_{L1}$ system and $\Psi {\left(N_{L2}\right)}_{N_{L2}N_{L1}}$ in the $N_{L2}N_{L1}$ system. Thus, if relative errors in DD phase observations are smaller than half the base unit—i.e., $\left|\varepsilon_{L1,L2}\right|<1.6$ mm—then the minima of the $\left|\Psi {\left(N_{L1}\right)}_{N_{L1}N_{L2}}\right|$ and $\left|\Psi {\left(N_{L2}\right)}_{N_{L2}N_{L1}}\right|$ functions indicate the searched ambiguities on the horizontal axis as, respectively: $N_{L1}$ using the minimum of the $\left|\Psi {\left(N_{L1}\right)}_{N_{L1}N_{L2}}\right|$ function and $N_{L2}$ using the minimum of the $\left|\Psi {\left(N_{L2}\right)}_{N_{L2}N_{L1}}\right|$ function. If correlated errors are equal to zero, in both of the above cases, approximate values of ${\tilde{N}}_{L1}$ and ${\tilde{N}}_{L2}$ must be at a distance smaller than 0.5$\lambda_{\Psi {\left(N_{L1}\right)}_{N_{L1}N_{L2}}}$ or 0.5 $\lambda_{\Psi {\left(N_{L2}\right)}_{N_{L2}N_{L1}}}$ from the real values (${\hat{N}}_{L1}$ and ${\hat{N}}_{L2}$), thus smaller than:

(39) $$\left|{\tilde{N}}_{L1}-{\hat{N}}_{L1}\right|<7.32630640273705\;m$$

and/or

(40) $$\left|{\tilde{N}}_{L2}-{\hat{N}}_{L2}\right|<7.32630640273705\;m$$

If, however, the absolute $\Delta_{\Psi {\left(N_{L1}\right)}_{N_{L1}N_{L2}}}$ or values are different from zero and larger than 1.6 mm, then ambiguity values will be located in different points than the minima of the $\left|\Psi {\left(N_{L1}\right)}_{N_{L1}N_{L2}}\right|$ and $\left|\Psi {\left(N_{L2}\right)}_{N_{L2}N_{L1}}\right|$ functions, and their selection will strongly depend on the real $\varepsilon_{L1,L2}$ values and from the approximate ${\tilde{N}}_{L1}$ and ${\tilde{N}}_{L2}$ values. 

Thus, knowing the true values of $\varepsilon_{L1,L2}$ errors, we are able to explicitly calculate the searched $N_{L1}$ and $N_{L2}$ ambiguities based on mathematical $\Psi {\left(N_{L1}\right)}_{N_{L1}N_{L2}}$ and/or $\Psi {\left(N_{L2}\right)}_{N_{L2}N_{L1}}$ functions, as within the range of 7.3263 m we can only obtain a single candidate for (${\hat{N}}_{L1},{\hat{N}}_{L2}$) ambiguity that satisfies the equations:

(41) $$\varepsilon_{L1,L2}=\Psi {\left(N_{L1}={\hat{N}}_{L1}\right)}_{N_{L1}N_{L2}}$$

(42) $$-\varepsilon_{L1,L2}=\Psi {\left(N_{L2}={\hat{N}}_{L2}\right)}_{N_{L2}N_{L1}}$$

However, in reality, we do not know the true value of $\varepsilon_{L1,L2}$ errors. Therefore, searching for unknown $N_{L1}$ and $N_{L2}$ ambiguities starts with the smallest values of the $\left|\Psi {\left(N_{L1}\right)}_{N_{L1}N_{L2}}\right|$ or $\left|\Psi {\left(N_{L2}\right)}_{N_{L2}N_{L1}}\right|$ functions in a certain, direct neighborhood of float solution (${\tilde{N}}_{L1}$ ${\tilde{N}}_{L2}$), with the ambiguities being selected in line with the following relation:

(43) $$\left|\Psi {\left(N_{L1}=N_{L1}^{I}\right)}_{N_{L1}N_{L2}}\right|<\left|\Psi {\left(N_{L1}=N_{L1}^{II}\right)}_{N_{L1}N_{L2}}\right|<\left|\Psi {\left(N_{L1}=N_{L1}^{III}\right)}_{N_{L1}N_{L2}}\right|\ldots \;\mathrm{or}/\mathrm{and}$$

(44) $$\left|\Psi N_{L2}={\left(N_{L2}^{I}\right)}_{N_{L2}N_{L1}}\right|<\left|\Psi {\left(N_{L2}=N_{L2}^{II}\right)}_{N_{L2}N_{L1}}\right|<\left|\Psi {\left(N_{L2}=N_{L2}^{III}\right)}_{N_{L2}N_{L1}}\right|\ldots$$

(45) $$\mathrm{because}\;\left|\Psi {\left(N_{L1}=N_{L1}^{i}\right)}_{N_{L1}N_{L2}}\right|=\left|\Psi {\left(N_{L2}=N_{L2}^{i}\right)}_{N_{L2}N_{L1}}\right|.$$

**Figure 6:**
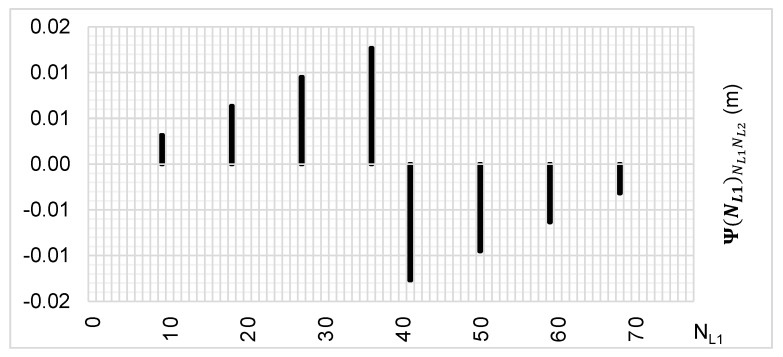
The area for seeking $N_{L1}$ ambiguities in phase measurements for the $\Psi {\left(N_{L1}\right)}_{N_{L1}N_{L2}}$ function and the $\varepsilon_{L1,L2}\subset \left(\;-14.3\;\mathrm{mm};+14.3\;\mathrm{mm}\right)$ value.

**Figure 7:**
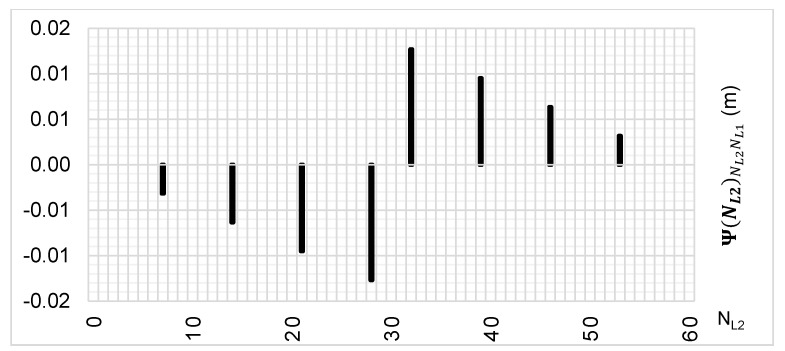
The area for seeking $N_{L2}$ ambiguities in phase measurements for the $\Psi {\left(N_{L2}\right)}_{N_{L2}N_{L1}}$ function and the $\varepsilon_{L2,L1}\subset \left(\;-14.3\;\mathrm{mm};+14.3\;\mathrm{mm}\right)$ value.

Let us thus assume that the $\varepsilon_{L1,L2}$ values for the $\Psi {\left(N_{L1}\right)}_{N_{L1}N_{L2}}$ function are in the range of ±14.3 mm—i.e., below 4.5 units $\Delta_{\Psi {\left(N_{L1}\right)}_{N_{L1}N_{L2}}}$. Then, our set of possible ambiguities is represented graphically in Figure 6 and Figure 7. Thus, the $\Psi {\left(N_{L1}\right)}_{N_{L1}N_{L2}}$ function indicates possible solutions for ambiguities with assumed $\varepsilon_{L1,L2}\in \left(-14.3\;\mathrm{mm};14.3\;\mathrm{mm}\right)$ relative errors, and these are the following values:

$$N_{L1}\in \left\{0;9;18;27;36;41;50;59;68;77\right\}$$

**Figure 8:**
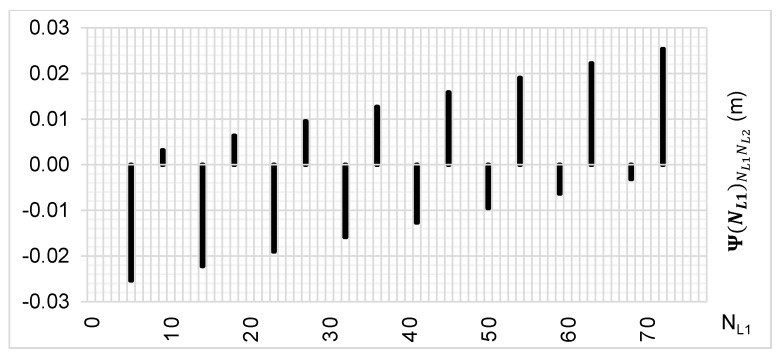
The area for seeking $N_{L1}$ ambiguities in phase measurements for the $\Psi {\left(N_{L1}\right)}_{N_{L1}N_{L2}}$ function and the $\varepsilon_{L1,L2}\subset \left(\;-27\;\mathrm{mm};\;+27\;\mathrm{mm}\right)\;$ value.

**Figure 9:**
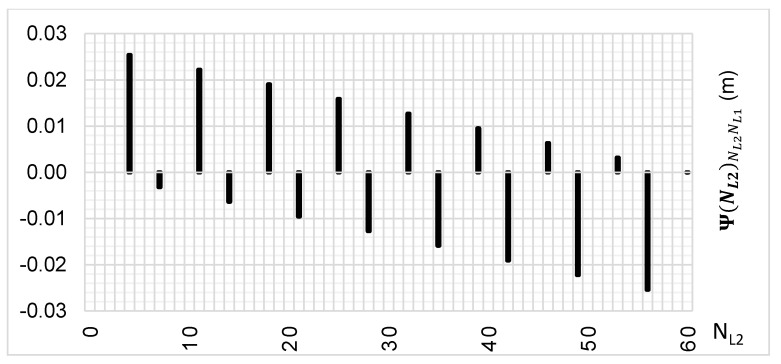
The area for seeking $N_{L2}$ ambiguities in phase measurements for the $\Psi {\left(N_{L2}\right)}_{N_{L2}N_{L1}}$ function and the $\varepsilon_{L2,L1}\subset \left(\;-27\;\mathrm{mm};\;+27\;\mathrm{mm}\right)\;$ value.

For comparison, search areas for $\varepsilon_{L1,L2}$ that are in the range of ±27 mm (i.e., up to 8.5 units) have been presented in Figure 8 and Figure 9. Then, the $\Psi {\left(N_{L1}\right)}_{N_{L1}N_{L2}}$ and $\Psi {\left(N_{L2}\right)}_{N_{L2}N_{L1}}$ functions indicated integer values for $N_{L1}$, in which the correct ambiguity can be located, and these are the following items:

$$N_{L1}\in \left\{0;5;9;14;18;23;27;32;36;41;45;50;54;59;63;68;72;77\right\}$$

$$N_{L2}\in \left\{0;4;7;11;14;18;21;25;28;32;35;39;42;46;49;53;56\right\}$$

If we assume, for example, that the values of relative errors in DD phase observations lie in the $\varepsilon_{L1,L2}\subset \left(0;\;27\;\mathrm{mm}\right)$ interval, then the $\Psi {\left(N_{L1}\right)}_{N_{L1}N_{L2}}$ function indicates integer $N_{L1}$ ambiguities every nine cycles, and these are the following values:

$$N_{L1}\in \left\{0;9;18;27;36;45;54;63;72;77\right\}$$

Similarly, if we assume, for example, that the values of relative errors in DD phase observations lie in the $\varepsilon_{L1,L2}\subset \left(-27\;\mathrm{mm};\;0\right)$ interval, then the $\Psi {\left(N_{L1}\right)}_{N_{L1}N_{L2}}$ function indicates integer $N_{L1}$ ambiguities every nine cycles, and these are the following values:

$$N_{L1}\in \left\{0;5;\;14;\;23;\;32;\;41;\;50;\;59;\;68;77\;\right\}$$

Thus, analysing only the $N_{L1}N_{L2}$, system for selected integer ambiguities $N_{L1}$, integer $N_{L2}$ values can be calculated with the use of the 12 and 15 formulas—i.e.,:

(46) $$N_{L2}={\left[\frac{60}{77}N_{L1}+{\tilde{N}}_{L2}-\frac{60}{77}{\tilde{N}}_{L1}\right]}_{r\mathrm{oundoff}}$$

Thus, for the $\varepsilon_{L1,L2}\subset \left(0;\;27\;\mathrm{mm}\right)\;$ interval and for the $\varepsilon_{L1,L2}\subset \left(-27\;\mathrm{mm};\;0\right)$ interval, these are [$N_{L1}$; $N_{L2}]$ integer sets:

$$\varepsilon_{L1,L2}\subset \left(0;\;+27\;\mathrm{mm}\right):\;[9;7],\;[18;14],\;[27;21],\;[36;28],\;[45;35],\;[54;42],\;[64;49],\;[72;56]$$

$$\varepsilon_{L1,L2}\subset \left(-27\;\mathrm{mm};\;0\right):\;[5;4],\;[14;11],\;[23;18],\;[32;25],\;[41;32],\;[50;39],\;[59;46],\;[68;53]$$

that differ by 9 and 7 cycles for $N_{L1}$ and $N_{L2}$, respectively.

**Figure 10:**
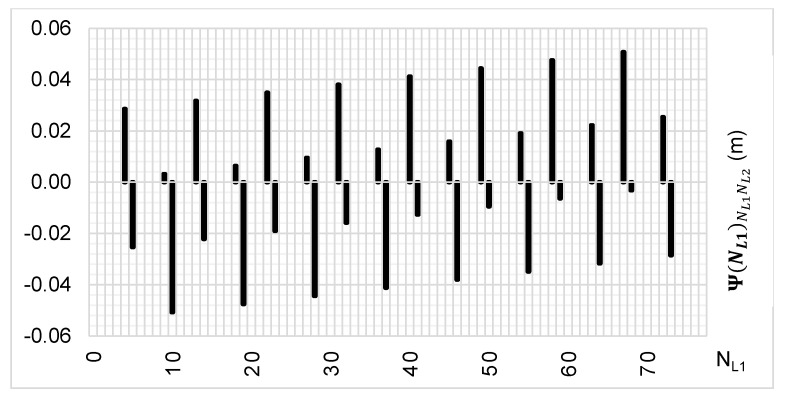
The area for seeking $N_{L1}$ ambiguities in phase measurements for the $\Psi {\left(N_{L1}\right)}_{N_{L1}N_{L2}}$ function and the $\varepsilon_{\left(L1,L2\right)}\subset \left(-52\;\mathrm{mm};\;+52\;\mathrm{mm}\right)$.

**Figure 11:**
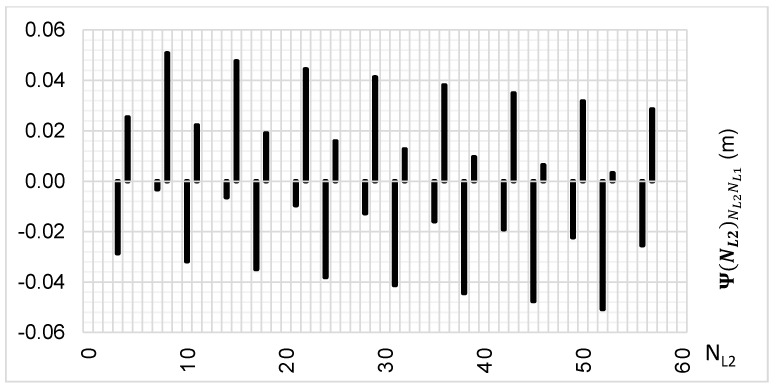
The area for seeking $N_{L2}$ ambiguities in phase measurements for the $\Psi {\left(N_{L2}\right)}_{N_{L2}N_{L1}}$ function and the $\varepsilon_{\left(L2,L1\right)}\subset \left(-52\;\mathrm{mm};\;+52\;\mathrm{mm}\right)$.

However, if we increase the range of relative errors—i.e., $\varepsilon_{L1,L2}\subset \left(-52\;\mathrm{mm};\;+52\;\mathrm{mm}\right)$—these will be the following proposals for $N_{L1}$ (Figure 10) and for $N_{L2}$ (Figure 11):

$$N_{L1}\in \left\{\begin{array}{c} 0;4;5;9;10;13;14;18;19;22;23;27;28;31;32;36;37;\\ 40;41;45;46;49;50;54;55;58;59;63;64;67;68;72;73;77 \end{array}\right\}$$

$$N_{L2}\in \left\{\begin{array}{c} 0;3;4;7;8;10;11;14;15;17;18;21;22;24;25;28;29\\ 31;32;35;36;38;39;42;43;45;46;49;50;52;53;56;57;60 \end{array}\right\}$$

The Figure 10 and Figure 11 present templates for possible ambiguities for relative errors in DD phase observations with the values up to ±52 mm. This range seems sufficient for determining ambiguities in real-life conditions for short baselines or when using data from virtual reference stations.

## 6. Numerical Example Using the PREFMAR Method and a Float Solution

As mentioned in the introduction, although there are numerous methods for determining ambiguity, the PREFMAR method can provide sets ambiguities for any pair of satellites without the VC matrix with a float solution. Therefore, the goal of this chapter is to present numerical calculations to indicate the most probable [$N_{L1,\;i}$, $N_{L2,\;i}]$ pairs that can later be used as input data for final ambiguity determination—i.e., validation. However, the PREFMAR allows for (mathematically) unambiguous determination of ambiguities based on known relative errors in DD phase observations—i.e., the $\varepsilon_{L1,L2}$ or $\varepsilon_{L1,L2}$ values (Equations (25) and (26)). In reality, it is unable to determine these values precisely, but it can be assumed that the search area starts with the $\varepsilon_{L1,L2}$ or $\varepsilon_{L1,L2}$ values with the smallest absolute values, which seems logical for short baselines. Thus, for calculations, we use real single epoch L1 and L2 of GPS data. For calculations we use values from the books [1,24]—i.e., approximate ${\tilde{N}}_{L1}$ and ${\tilde{N}}_{L2}$ values and elements of the variance-covariance matrix $Q_{\tilde{N}}$ of a float solution:

(47) $$\tilde{N}=\left[\begin{array}{c} {\tilde{N}}_{L1}\\ {\tilde{N}}_{L2} \end{array}\right]=\left[\begin{array}{c} 1.05\\ 1.30 \end{array}\right]\;\mathrm{and}\;Q_{\tilde{N}}=\left[\begin{array}{cc} 53.40 & 38.40\\ 38.40 & 28.00 \end{array}\right].$$

Sets of ambiguities can be determined both with the use of the $\Psi {\left(N_{L1}\right)}_{N_{L1}N_{L2}}$ function and the $\Psi {\left(N_{L2}\right)}_{N_{L2}N_{L1}}$ function. However, if we decide to use the $\Psi {\left(N_{L1}\right)}_{N_{L1}N_{L2}}$ function, the search area can be determined as $N_{L1}\subset$<$integer[1,05-\sqrt{53.4}$]; $integer\left[\;1.05+\sqrt{53.4}\right]>$—i.e., $N_{L1}\subset$<−6; 8>. Thus, let us present the necessary calculations in the form of Table 1 where elements of the ${\tilde{N}}_{L2}$ columns are determined based on an expression for integer and subsequent $N_{L1}$ values, in the ${\tilde{N}}_{L1}$ and ${\tilde{N}}_{L2}$ neighbourhoods, using the following formula:

(48) $${\tilde{N}}_{L2}=\frac{60}{77}*N_{L1}+1.30-\frac{60}{77}*1.05$$

and the values of the $\Psi {\left(N_{L1}\right)}_{N_{L1}N_{L2}}$ function are calculated with the formula:

(49) $$\Psi {\left(N_{L1}\right)}_{N_{L1}N_{L2}}=\lambda_{L2}\left(\frac{60}{77}N_{L1}+1.30-\frac{60}{77}*1.05-{\left[\frac{60}{77}N_{L1}+1.30-\frac{60}{77}*1.05\right]}_{\mathrm{roundoff}}\right)$$

However, if we decide to use the $\Psi {\left(N_{L2}\right)}_{N_{L2}N_{L1}}$ function, the search area can be determined as $N_{L2}\subset$ < $integer[1.30-\sqrt{28}$]; $integer\left[\;1.30+\sqrt{28}\right]>$—i.e., $N_{L2}\subset$ < −4; 7>. Detailed calculations are presented in Table 2.

**Figure 12:**
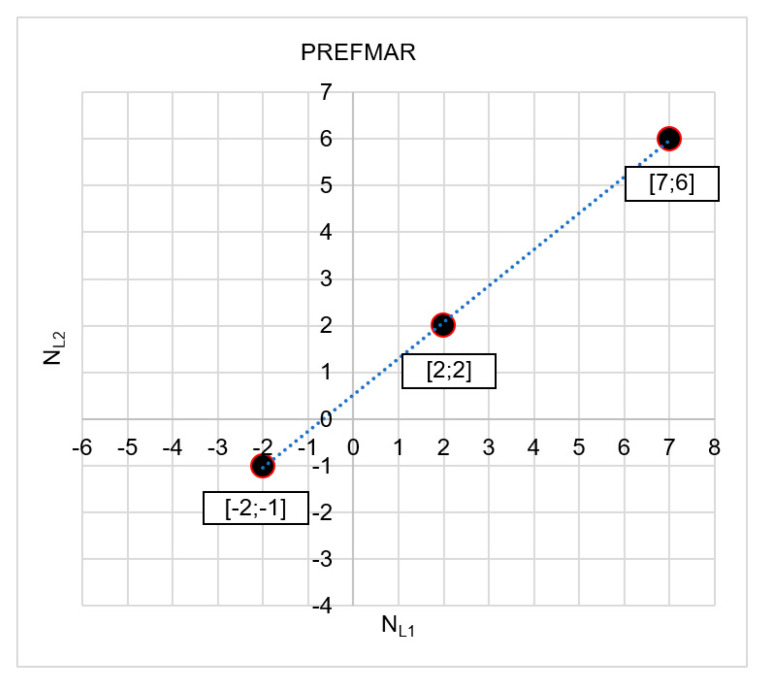
The searched sets of ambiguities in the $N_{L1}N_{L2}$ system for $\left|\Psi {\left(N_{L1}\right)}_{N_{L1}N_{L2}}\right|<3.5\;\mathrm{cm}$ and for $\sigma_{P}=1.4\;m$.

The most probable solution includes the set of ambiguities: $N_{L1}=2$ and $N_{L2}=2$ where the functions achieved the smallest absolute values—i.e., $\left|\Psi {\left(N_{L1}=2\right)}_{N_{L1}N_{L2}}\right|=\;\left|\Psi {\left(N_{L2}=2\right)}_{N_{L2}N_{L1}}\right|=0.010$. Thus, based on two functions, we received the same results (Figure 12) because

(50) $$\begin{array}{c} \left|\Psi {\left(N_{L1}^{I}=2\right)}_{N_{L1}N_{L2}}=0,010\right|<\left|\Psi {\left(N_{L1}^{II}=7\right)}_{N_{L1}N_{L2}}=-0.015\right|\\ <\left|\Psi {\left(N_{L1}^{III}=-2\right)}_{N_{L1}N_{L2}}=-0.019\right| \end{array}$$

and

(51) $$\begin{array}{c} \left|\Psi {\left(N_{L2}^{I}=2\right)}_{N_{L2}N_{L1}}=-0.010\right|<|\Psi {\left(N_{L2}^{II}=6\right)}_{N_{L2}N_{L1}}=0.015|\\ <\left|\Psi {\left(N_{L2}^{III}=-1\right)}_{N_{L2}N_{L1}}=0.019\right| \end{array}$$

Only three sets have been presented in Figure 12: I[2;2]; II[7;6]; and III[−2;−1], as these are the proposals with the assumption that relative errors in DD phase observations are smaller than 3.5 cm. It should also be noted that the proposed most likely values [$N_{L1}$;$\;N_{L2}]$ are also consistent with wide lane ($N_{WL}$) ambiguities, because $N_{WL}\left(I\right)=2-2=0$; $N_{WL}\left(II\right)=7-6=1;$ and $N_{WL}\left(III\right)=-2+1=-1$. Therefore, the indicated sets of ambiguity $N_{WL}$ with values 0, 1 and −1 are the most probable, because they represent neighboring and subsequent values of wide lane ambiguities [−1;0;1] in the immediate vicinity of the float solution.

**Figure 13:**
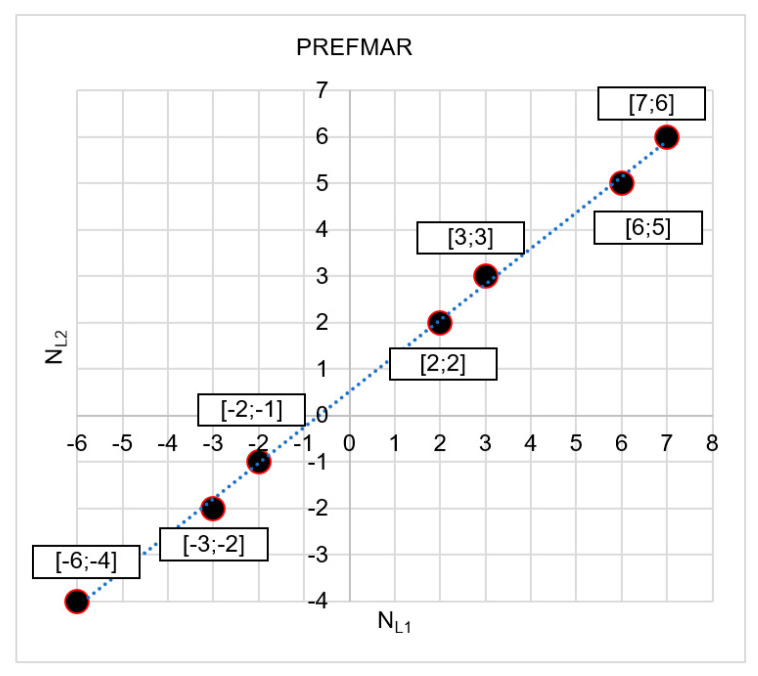
The searched sets of ambiguities in the $N_{L1}N_{L2}$ system for $\left|\Psi {\left(N_{L1}\right)}_{N_{L1}N_{L2}}\right|<6\;\mathrm{cm}$ and for $\sigma_{P}=1.4\;m$.

If we assume that relative errors in phase measurements for DD observations are smaller than 6 cm, the number of ambiguities increases, Figure 13.

Please note, however, the phase errors in DD measurements reaching 6 cm are quite large and can occur in the case of interferences in satellite signals or in the case of longer vectors. In such cases, we strive to increase code measurement accuracy to reduce the search area as far as possible. If, for instance, in the example presented above, we achieve a code measurement accuracy down to ca. 0.5 m, then we only have two ambiguity candidates—I[2;2]; II[3;3]—and even with the assumption that relative errors in phase measurements for DD observations—i.e., the values of the $\left|\Psi {\left(N_{L1}\right)}_{N_{L1}N_{L2}}\right|$ or $\left|\Psi {\left(N_{L2}\right)}_{N_{L2}N_{L1}}\right|$ functions are below 6.4 cm (see Table 1 and Table 2). 

**Figure 14:**
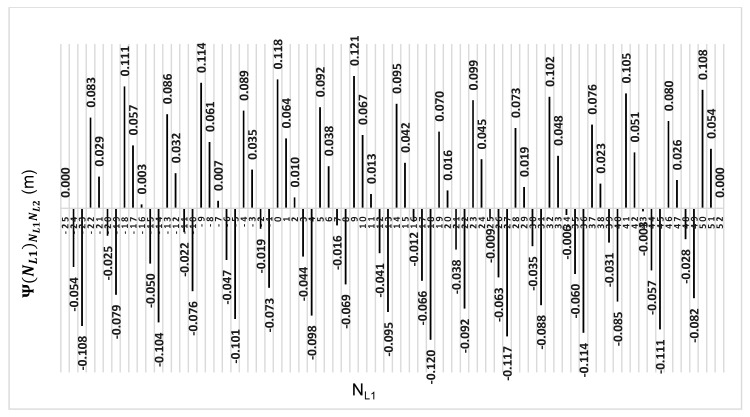
Behavior of the full period of the $\Psi {\left(N_{L1}\right)}_{N_{L1}N_{L2}}$ function, for ${\tilde{N}}_{L1}=1,05$; ${\tilde{N}}_{L2}=1.30$, and for $\left|\Psi {\left(N_{L1}\right)}_{N_{L1}N_{L2}}\right|<0.5\lambda_{L2}.$

Behavior of the full period of the $\Psi {\left(N_{L1}\right)}_{N_{L1}N_{L2}}$ function for input data has been presented in Figure 14, and below we presented precisely calculated minima of this function— i.e., $\left|\Psi {\left(N_{L1}=-25\right)}_{N_{L1}N_{L2}}\right|$ and $\left|\Psi {\left(N_{L1}=52\right)}_{N_{L1}N_{L2}}\right|$ based on the formula:

(52) $$\begin{array}{cc} \left|\Psi {\left(N_{L1}\right)}_{N_{L1}N_{L2}}\right| & =\lambda_{L2}\left|{\tilde{N}}_{L2}-{\left[{\tilde{N}}_{L2}\right]}_{\mathrm{roundoff}}\right|\\ & =\lambda_{L2}\left|a_{L1,L2}N_{L1}+b_{L1,L2}-{\left[a_{L1,L2}N_{L1}+b_{L1,L2}\right]}_{\mathrm{roundoff}}\right|\\ & =\lambda_{L2}\left|a_{L1,L2}N_{L1}+{\tilde{N}}_{L2}-a_{L1,L2}{\tilde{N}}_{L1}-{\left[a_{L1,L2}N_{L1}+{\tilde{N}}_{L2}-a_{L1,L2}{\tilde{N}}_{L1}\right]}_{\mathrm{roundoff}}\right| \end{array}$$

thus:

(53) $$\begin{array}{c} \left|\Psi {\left(N_{L1}=-25\right)}_{N_{L1}N_{L2}}\right|=\\ |0.7792207792208*\left(-25\right)+1.30-0.7792207792208*1.05-\\ {\left[0.7792207792208*\left(-25\right)+1.30-0.7792207792208*1.05\right]}_{\mathrm{roundoff}}|*\lambda_{L2}=\\ |-18.998701—18.998701{]}_{\mathrm{roundoff}}*\lambda_{L2}=0.001299\lambda_{L2}=0.00032\;m \end{array}$$

(54) $$\begin{array}{c} |\Psi {\left(N_{L1}=52\right)}_{N_{L1}N_{L2}}|=\\ |0.7792207792208*52+1.30-0.7792207792208*1.05-[0.7792207792208*52+\\ 1.30-0.7792207792208*1.05{]}_{\mathrm{roundoff}}|*\lambda_{L2}=\left|41.001299-{\left[41.001299\right]}_{\mathrm{roundoff}}\right|*\lambda_{L2}=\\ 0.001299\lambda_{L2}=0.00032\;m \end{array}$$

i.e.,

(55) $$\left|\Psi {\left(N_{L1}=-25\right)}_{N_{L1}N_{L2}}\right|=\left|\Psi {\left(N_{L1}=52\right)}_{N_{L1}N_{L2}}\right|$$

Generally, for the $N_{L1}N_{L2}$ system, the search area takes the form of a parallelogram with sides identical to those described above. The short sides of this parallelogram are parallel to the vertical lines of the system $N_{L1}N_{L2}$—i.e., to the $N_{L2}$ axis. The longer side of this parallelogram is slanted with respect to the $N_{L2}$ axis under and $\alpha_{L1,L2}=tan^{-1}\left(\frac{60}{77}\right)=37.92646233^{{}^{\circ}}$ angle and the geometrical centre of the search area is located in point $({\tilde{N}}_{L1}$, ${\tilde{N}}_{L2})$. To precisely determine the search area for the PREFMAR method, it is thus necessary to know the accuracy of $\sigma_{P}$ code measurements and allowable values of relative errors that can take maximum values of $0.5\lambda_{L2}$. 

In the end, it should be noted that the most probable integer candidates calculated by the PREFMAR—i.e., $N_{L1}=2$ and $N_{L2}=2$ are the same as the LAMBDA (Least-squares AMBiguity Decorrelation Adjustment) result [24].

## 7. Discussion of the PREFMAR’s Efficiency in Terms of Initialization and Reinitialization Based on Real Positioning Data Using L1-L2 GPS Measurements.

**Figure 15:**
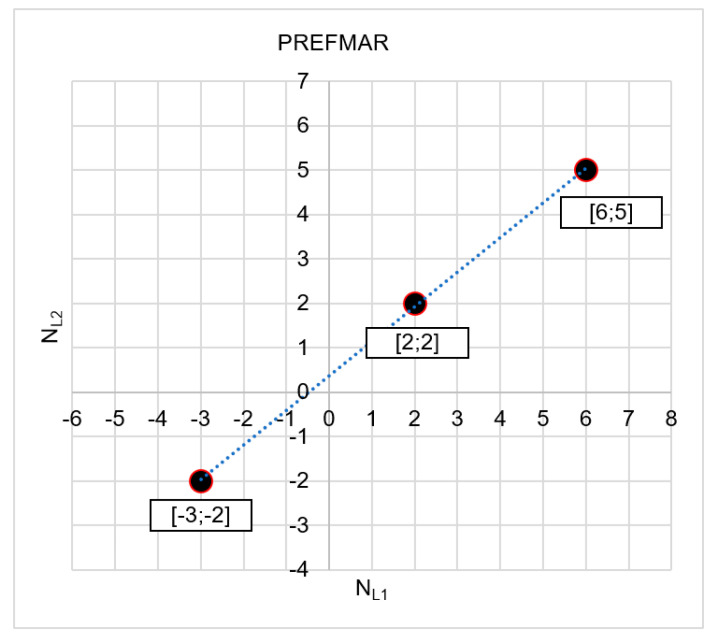
The searched sets of ambiguities in the $N_{L1}N_{L2}$ system for $\Psi {\left(N_{L1}\right)}_{N_{L1}N_{L2}}\subset \left(\;0\;;6\;\mathrm{cm}\right)$ and for $\sigma_{P}=1.4\;m$.

**Figure 16:**
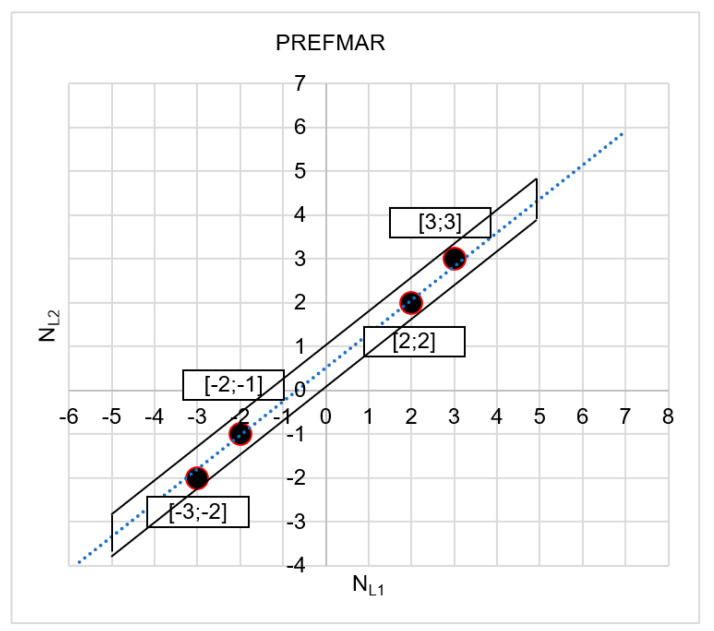
The search area of ambiguities in the $N_{L1}N_{L2}$ system for $\left|\Psi {\left(N_{L1}\right)}_{N_{L1}N_{L2}}\right|<6\;\mathrm{cm}$, where $N_{L1}\subset \langle -5;+5\rangle$.

The PREFMAR method uses the functions described above: $\Psi {\left(N_{L1}\right)}_{N_{L1}N_{L2}}$ and/or $\Psi {\left(N_{L2}\right)}_{N_{L2}N_{L1}}$ to indicate the most likely sets of ambiguities for L1-L2 GPS measurements. The functions presented relate to relative errors of DD phase observations. Thus, these errors can be both positive or negative. Therefore, function values $\Psi {\left(N_{L1}\right)}_{N_{L1}N_{L2}}$ or $\Psi {\left(N_{L2}\right)}_{N_{L2}N_{L1}}$ can more precisely determine the search area in case of reinitialization of ambiguities—i.e., where we have already determined our ambiguities but lost contact with some or all satellites for various reasons. This process of ambiguity reinitialization is more common in practice than the initialization process—i.e., the first determination of sets of ambiguities for a given vector. Thanks to the discovered properties of the developed mathematical functions, used in the PREFMAR method, and using relative errors in the reinitialization process, we are able to reduce the number of combinations in the validation process even several dozen times. For example, let us imagine that in the above example we would reinitialize the ambiguity in time (t), having previously determined ambiguities in time (t−1)—i.e., we have at our disposal values of the function $\Psi {\left(N_{L1}\right)}_{N_{L1}N_{L2}}\left(t-1\right)$. For short vectors, several kilometers in length, it is sufficient that only the information whether the value of function $\Psi {\left(N_{L1}\right)}_{N_{L1}N_{L2}}\left(t-1\right)$ is positive or negative. Suppose that $\Psi {\left(N_{L1}\right)}_{N_{L1}N_{L2}}\left(t-1\right)>0$, this means that in the reinitialization process in the example shown above, our most probable sets of ambiguities will be as follows: (Figure 15) I[2;2]; II[−3;−2]; and III[6;5], for the function values $\left|\Psi {\left(N_{L1}\right)}_{N_{L1}N_{L2}}\right|<6\;\mathrm{cm}$. Thus, during the re-initialization process, we obtained a significant reduction in possible sets of ambiguities from 7 pairs to 3 pairs. If we assume that we have four satellites and perform validation with 7 pairs of possible sets of ambiguities for four satellites we obtain 7^3^ = 343 combinations. In the proposed reinitialization process, we have only 3^3^ = 27 combinations. Therefore, using the described properties of mathematical functions, the PREFMAR method enables immediate reinitialization of ambiguities for single observation epochs for different baseline lengths. Paradoxically, the length of baselines is not a problem in the reinitialization process, as larger relative errors will be more reliable in the process of determining the search area. Therefore, the search area of the PREFMAR method can be presented in several variants, depending on the length of the vector and with regard whether we are dealing with initialization or reinitialization. In the presented calculation example, on the basis of the variance–covariance matrix, we can see that we have quite a large error in code measurements—i.e., about 1.4 m. The current capabilities of GNSS receivers allow quite good code accuracy, especially when using the Kalman filter, and obtaining code accuracy below the wavelength $N_{w}=0.86$ m is not a problem. This means that for short baselines, it is enough to give four sets of ambiguities, among which the value searched for should be included (Figure 16).

This means that for the frequency L1-L2, we should try to obtain code accuracy of less than 0.86 m, and then for short vectors we give only four sets of ambiguities—i.e., I [2 ,2]; II[−2,−1]; III[−3,−2]; IV[3,3]. This method has been proven to be relevant for relative errors of phase observations below 6 cm; which is absolutely sufficient for baselines of several kilometers or for baselines in relation to virtual reference stations (VRS).

**Figure 17:**
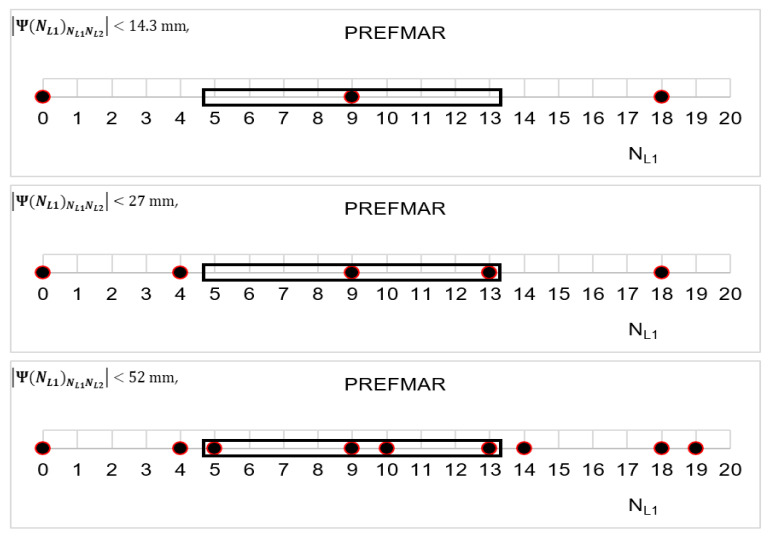
The search area of ambiguities in the $N_{L1}N_{L2}$ system for: $\left|\Psi {\left(N_{L1}\right)}_{N_{L1}N_{L2}}\right|<14.3\;\mathrm{mm}$; $\left|\Psi {\left(N_{L1}\right)}_{N_{L1}N_{L2}}\right|<27\;\mathrm{mm}$; $\left|\Psi {\left(N_{L1}\right)}_{N_{L1}N_{L2}}\right|<52\;\mathrm{mm}$; and for $\sigma_{P}<0.86\;m$.

The dependence of the number of ambiguity sets on the accuracy of code measurements below 0.86 m using the PREFMAR method is shown in Figure 17.

In Figure 17, we can see that in the case of relative errors in the range $\varepsilon_{L1,L2}\subset (-14.3\;\mathrm{mm};\;+14.3\;\mathrm{mm}$), we have only one set of ambiguity. For relative errors in the range $\varepsilon_{L1,L2}\subset (-27\;\mathrm{mm};\;+27\;\mathrm{mm}$), we have two sets of ambiguity. However, for the relative errors $\varepsilon_{L1,L2}\subset \left(-52\;\mathrm{mm};\;+52\;\mathrm{mm}\right),$ we have four sets. Note that whenever we obtain an even number of ambiguity sets, half of the sets are determined by positive values of the function $\Psi {\left(N_{L1}\right)}_{N_{L1}N_{L2}}$, and the other half by negative values.

In addition, in the case of reinitialization in time (t), we use the function values from the time before the disturbance in the reception of the continuity of phase signals—i.e., from time (t−η) as follows:

(56) $$\Psi {\left(N_{L1}\right)}_{N_{L1}N_{L2}}\left(t\right)=\Psi {\left(N_{L1}\right)}_{N_{L1}N_{L2}}\left(t\right)-\Psi {\left(N_{L1}={\hat{N}}_{L1}\right)}_{N_{L1}N_{L2}}\left(t-\eta \right)$$

(57) $$\Psi {\left(N_{L2}\right)}_{N_{L2}N_{L1}}\left(t\right)=\Psi {\left(N_{L2}\right)}_{N_{L2}N_{L1}}\left(t\right)-\Psi {\left(N_{L2}={\hat{N}}_{L2}\right)}_{N_{L2}N_{L1}}\left(t-\eta \right)$$

where η represents the time delay—i.e., the initialization time minus the time of the last epoch with fixed ambiguities: $\eta =\left(t\right)-{\left(t\right)}^{Fixed}$

For the DD observation at time (t) during reinitialization, we can, therefore, write the following functions:

(58) $$\begin{array}{cc} \Psi {\left(N_{L1}\right)}_{N_{L1}N_{L2}}\left(t\right)= & \lambda_{L2}\left({\tilde{N}}_{L2}-{\left[{\tilde{N}}_{L2}\right]}_{\mathrm{roundoff}}\right)-\Psi {\left(N_{L1}={\hat{N}}_{L1}\right)}_{N_{L1}N_{L2}}\left(t-\eta \right)=\\ \lambda_{L2}(\frac{60}{77}N_{L1}+ & \left(\phi_{L2}-\frac{\omega }{\lambda_{L2}}\right)-\frac{60}{77}\left(\phi_{L1}-\frac{\omega }{\lambda_{L1}}\right)\\ & -{\left[\frac{60}{77}N_{L1}+\left(\phi_{L2}-\frac{\omega }{\lambda_{L2}}\right)-\frac{60}{77}\left(\phi_{L1}-\frac{\omega }{\lambda_{L1}}\right)\right]}_{\mathrm{roundoff}})\\ & -\Psi {\left(N_{L1}={\hat{N}}_{L1}\right)}_{N_{L1}N_{L2}}\left(t-1\right) \end{array}$$

(59) $$\begin{array}{cc} \Psi {\left(N_{L2}\right)}_{N_{L2}N_{L1}}\left(t\right)= & \lambda_{L1}\left({\tilde{N}}_{L1}-{\left[{\tilde{N}}_{L1}\right]}_{\mathrm{roundoff}}\right)-\Psi {\left(N_{L2}={\hat{N}}_{L2}\right)}_{N_{L2}N_{L1}}\left(t-\eta \right)=\\ \lambda_{L1}(\frac{77}{60}N_{L2}+ & \left(\phi_{L1}-\frac{\omega }{\lambda_{L1}}\right)-\frac{77}{60}\left(\phi_{L2}-\frac{\omega }{\lambda_{L2}}\right)\\ & -{\left[\frac{77}{60}N_{L2}+\left(\phi_{L1}-\frac{\omega }{\lambda_{L1}}\right)-\frac{77}{60}\left(\phi_{L2}-\frac{\omega }{\lambda_{L2}}\right)\right]}_{\mathrm{roundoff}})\\ & -\Psi {\left(N_{L2}={\hat{N}}_{L2}\right)}_{N_{L2}N_{L1}}\left(t-1\right) \end{array}$$

where: ${\hat{N}}_{L1}$, ${\hat{N}}_{L2}$ represent fixed ambiguities in the time (t−η).

**Figure 18:**
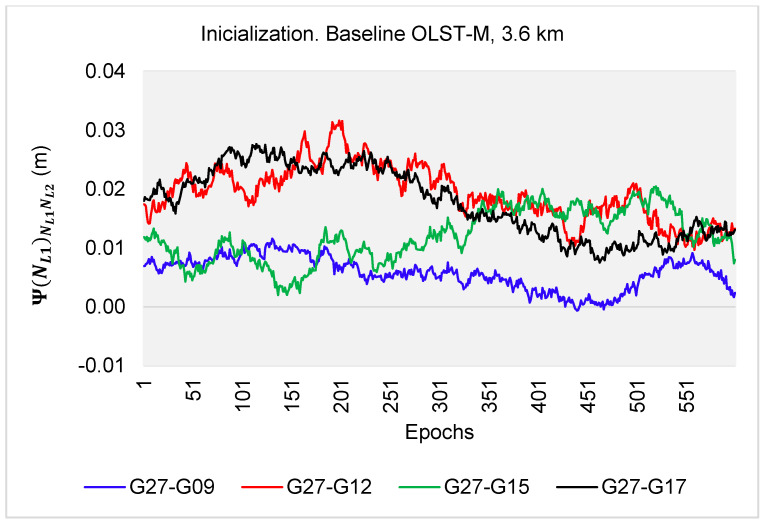
Values of the function $\Psi {\left(N_{L1}\right)}_{N_{L1}N_{L2}}\left(t\right)$ in real L1-L2 measurements for fixed ambiguities, for the 3.6 km baseline.

The developed algorithms were tested on real L1-L2 observations, for a 10-min session with an interval of 1 s, and a baseline length of 3.6 km. The L1-L2 GPS measurements were performed with the Topcon HiperPro (M) receiver on 13rd August 2010, from 10:30:00 to 10:40:00 UTC, with reference to the OLST reference station. There were five GPS satellites available above the horizon: G09, G12, G15, G17 and G27, for which the following double-difference (DD) observations were created: G27-G09, G27-G12, G27-G15 and G27-G17. For every double-difference carrier phase observation and correctly determined ambiguities, the relative errors $\varepsilon_{L1,L2}$ between L1 and L2 double-difference measurements were below 52 mm (see Figure 18). The ambiguities from the first range $\varepsilon_{L1,L2}\subset \left(-14.3\;\mathrm{mm};\;+14.3\;\mathrm{mm}\right)$ were for 1317 (55%) DD phase observations, in the second range $\varepsilon_{L1,L2}\subset \left(14.3\;\mathrm{mm};\;27\;\mathrm{mm}\right)$ there were 1039 (43%) DD phase observations. However, for the range $\varepsilon_{L1,L2}\subset \left(27\;\mathrm{mm};\;52\;\mathrm{mm}\right),$ there were only 44 DD phase observations (2%). Detailed data on the number of DD observations depending on the value of the relative errors $\varepsilon_{L1,L2}$ are presented in Table 3.

**Figure 19:**
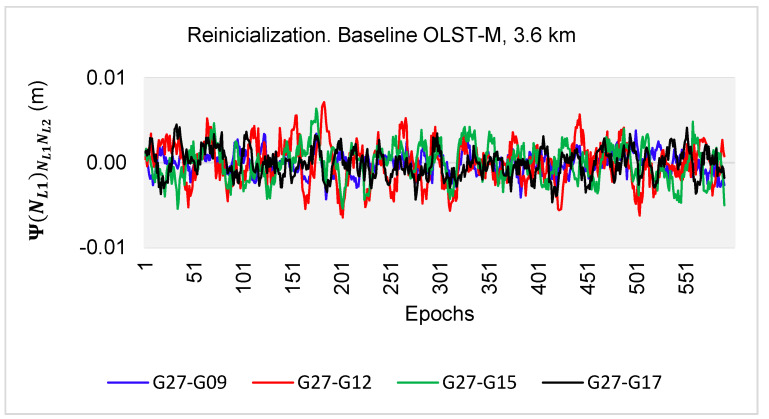
Values of the function $\Psi {\left(N_{L1}\right)}_{N_{L1}N_{L2}}\left(t-\eta \right)$ in the reinitialization approach for L1-L2 measurements based on the PREFMAR, for η=10 secund.

In the second stage of the numerical tests, in the case of reinitialization at (t−η)=10 s, the values of the function $\Psi {\left(N_{L1}\right)}_{N_{L1}N_{L2}}\left(t\right)$ were obtained in the range −14.3–+14.3 mm for all DD observations (Figure 19), and, therefore, all (100%) first ambiguity sets at reinitialization determined by the PREFMAR method were the searched unknowns.

The properties of the functions $\Psi {\left(N_{L1}\right)}_{N_{L1}N_{L2}}\left(t\right)$ or $\Psi {\left(N_{L2}\right)}_{N_{L2}N_{L1}}\left(t\right)$ and the stability of the relative errors during the measurements enable to instantaneous ambiguity reinitialization because the search area is the same as for ultra-short baselines. Then, for the float solution, with the use of code measurements P1 and P2 below 0.86 m, and for the relative errors in the range $\varepsilon_{L1,L2}\subset \left(-14.3\;\mathrm{mm};\;+14.3\;\mathrm{mm}\right)$, we obtain only one set of ambiguities (Figure 18). Therefore, the PREFMAR allows instantaneous ambiguity reinitialization for L1-L2 GPS measurements, for short as well for longer baselines.

## 8. Summary and Conclusions

This work presents the new PREFMAR method for determining ambiguities in phase measurements for single measurement epochs, for GPS observations performed at L1 and L2 frequencies. Its efficiency mostly depends on the values of relative errors in DD phase observations. Ambiguity is selected based on a proprietary search function that uses the correlation between precisely determined ambiguity values depending on the frequency of satellite signals. The PREFMAR method allows determining ambiguities for individual measurement epochs without using a variance–covariance matrix with a float solution. For relative errors of L1 and L2  carrier phase measurements $\varepsilon_{L1,L2}\subset \left(-14.3\;\mathrm{mm};\;+14.3\;\mathrm{mm}\right),$ the search area is as if we were using frequencies for a wavelength equal to 1.73 m (see Figure 17). Thus, two neighboring ambiguities cover an area equivalent to an 18-cycle range $N_{L1}$ and 14-cycle range $N_{L2}$ in the $N_{L1}N_{L2}$ system. This is a remarkable feature compared to the L1-L5 combination.

In the case of relative errors of  L1 and L2 carrier phase measurements $\varepsilon_{L1,L2}\subset \left(\;-27\;\mathrm{mm};\;+27\;\mathrm{mm}\right),$ the search area is as if we were using frequencies for a wavelength equal to 0.86 m. Furthermore, for these relative errors, the wide lane ambiguity is unambiguously recalculated to $N_{L1}$ and $N_{L2}$.

The developed PREFMAR method indicates ambiguity sets ($N_{L1}$, $N_{L2}$) for L1 and L2 frequencies, using the functions $\Psi {\left(N_{L1}\right)}_{N_{L1}N_{L2}}$ or $\Psi {\left(N_{L2}\right)}_{N_{L2}N_{L1}}$, from which mathematical equations have been derived and described in detail in this work. The identification of the ambiguity sets ($N_{L1}$, $N_{L2}$) can be performed if we have the following input data:

- GPS float solution;
- Global code GPS/GNSS solution XYZ (differential or relative) and single-epoch double-differenced L1-L2 GPS measurements;
- A single-epoch of double-differenced L1-L2 GPS measurements.

Note that for case III, the PREFMAR method shows ambiguities even for a single DD observation, for any pair of satellites, but the accuracy of the code measurements is of key importance as we use double-difference observations from only two satellites. However, for much more accurate P5 code measurements transmitted at the L5 frequency, the III approach can be effectively used in L1-L5 positioning. The approach II seems to be the most universal, because in the global XYZ solution we can use any configuration of GNSS satellites with the most accurate code measurements and Kalman filter, which significantly increases the accuracy of the solution of the approximate XYZ position, both in static and kinematic positioning. Based on preliminary numerical results, the PREFMAR allows instantaneous ambiguity reinitialization if all satellites lost contact with a GNSS antenna.

## Figures and Tables

**Table 1:** The set of integer candidates based on the $\Psi {\left(N_{L1}\right)}_{N_{L1}N_{L2}}$ function, where $N_{L1}\subset \langle -6;+8\rangle$.

$N_{L1}$	${\tilde{N}}_{L2}$	$\Psi {\left(N_{L1}\right)}_{N_{L1}N_{L2}}\;[m]$	*No. Sol.*	$N_{L2}={\left[{\tilde{N}}_{L2}\right]}_{\mathrm{roundoff}}$
−6	−4.193	−0.047		−4
−5	−3.414	−0.101		−3
−4	−2.635	0.089		−3
−3	−1.855	0.035		−2
−2	−1.076	−0.019	III	−1
−1	−0.297	−0.073		0
0	0.482	0.118		0
1	1.261	0.064		1
2	2.041	0.010	I	2
3	2.820	−0.044		3
4	3.599	−0.098		4
5	4.378	0.092		4
6	5.158	0.038		5
7	5.937	−0.015	II	6
8	6.716	−0.069		7

**Table 2:** The set of integer candidates based on the function $\Psi {\left(N_{L2}\right)}_{N_{L2}N_{L1}}$ where $N_{L2}\subset \langle -4;+7\rangle$.

$N_{L2}$	${\tilde{N}}_{L1}$	$\Psi {\left(N_{L2}\right)}_{N_{L2}N_{L1}}\left[m\right]$	*No. Sol.*	$N_{L1}={\left[{\tilde{N}}_{L1}\right]}_{roundoff}$
−4	−5.752	0.047		−6
−3	−4.469	−0.089		−4
−2	−3.186	−0.035		−3
−1	−1.902	0.019	III	−2
0	−0.619	0.073		−1
1	0.665	−0.064		1
2	1.948	−0.010	I	2
3	3.231	0.044		3
4	4.515	−0.092		5
5	5.798	−0.038		6
6	7.081	0.015	II	7
7	8.365	0.069		8

**Table 3:** The number of double-difference (DD) observations in the respective ranges of the relative errors of phase observations.

	$\varepsilon_{L1,L2}<14.3\;mm$	$\varepsilon_{L1,L2}\subset \left(14.3\;mm;27\;mm\right)$	$\varepsilon_{L1,L2}\subset \left(27\;mm;52\;mm\right)$
G27-G09	600	0	0
G27-G12	103	465	32
G27-G15	401	199	0
G27-G17	213	375	12
